# Supplementary material for: Saccharomyces boulardii (CNCM I-745) improves intestinal damage in sepsis by remodeling intestinal flora structure
Source: Front Cell Infect Microbiol. 2025 Jul 31;15:1602792. doi: 10.3389/fcimb.2025.1602792 (PMC12350329; doi:10.3389/fcimb.2025.1602792)
Supplement: Supplementary file 1 [file DataSheet1.zip › summary/summary/8_Advanced_analyse/2_PICRUSt2_result/METACYC/Group.AW4_vs_AW0/PICRUSt2_METACYC_Group.AW4_vs_AW0_diff.pdf]

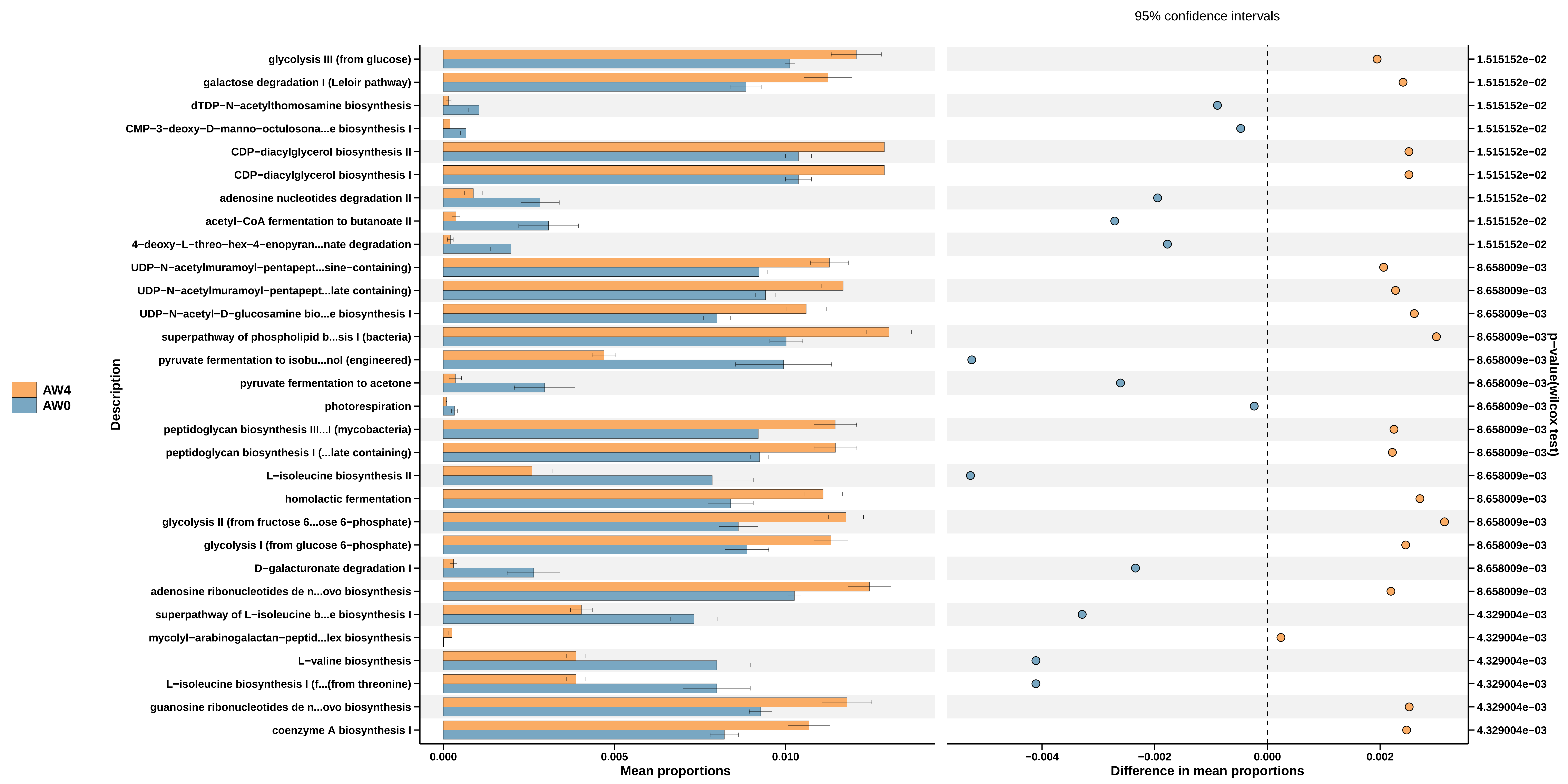
95% confidence intervals
glycolysis III (from glucose)
galactose degradation I (Leloir pathway)
dTDP−N−acetylthomosamine biosynthesis
CMP−3−deoxy−D−manno−octulosona...e biosynthesis I
CDP−diacylglycerol biosynthesis II
CDP−diacylglycerol biosynthesis I
adenosine nucleotides degradation II
acetyl−CoA fermentation to butanoate II
4−deoxy−L−threo−hex−4−enopyran...nate degradation
UDP−N−acetylmuramoyl−pentapept...sine−containing)
UDP−N−acetylmuramoyl−pentapept...late containing)
UDP−N−acetyl−D−glucosamine bio...e biosynthesis I
superpathway of phospholipid b...sis I (bacteria)
pyruvate fermentation to isobu...nol (engineered)
pyruvate fermentation to acetone
photorespiration
peptidoglycan biosynthesis III...I (mycobacteria)
peptidoglycan biosynthesis I (...late containing)
L−isoleucine biosynthesis II
homolactic fermentation
glycolysis II (from fructose 6...ose 6−phosphate)
glycolysis I (from glucose 6−phosphate)
D−galacturonate degradation I
adenosine ribonucleotides de n...ovo biosynthesis
superpathway of L−isoleucine b...e biosynthesis I
mycolyl−arabinogalactan−peptid...lex biosynthesis
L−valine biosynthesis
L−isoleucine biosynthesis I (f...(from threonine)
guanosine ribonucleotides de n...ovo biosynthesis
coenzyme A biosynthesis I
Description
AW4
AW0
0.000
0.005
0.010
Mean proportions
−0.004
−0.002
0.000
0.002
Difference in mean proportions
p−value(wilcox test)
1.515152e−02
1.515152e−02
1.515152e−02
1.515152e−02
1.515152e−02
1.515152e−02
1.515152e−02
1.515152e−02
1.515152e−02
8.658009e−03
8.658009e−03
8.658009e−03
8.658009e−03
8.658009e−03
8.658009e−03
8.658009e−03
8.658009e−03
8.658009e−03
8.658009e−03
8.658009e−03
8.658009e−03
8.658009e−03
8.658009e−03
8.658009e−03
4.329004e−03
4.329004e−03
4.329004e−03
4.329004e−03
4.329004e−03
4.329004e−03
